# *QuickStats:* Number of Emergency Department Visits*^,^^†^ for Substance Abuse or Dependence**^§^** per 10,000 Persons Aged ≥18 Years, by Age Group — United States, 2008–2009 and 2016–2017

**DOI:** 10.15585/mmwr.mm6850a7

**Published:** 2019-12-20

**Figure:**
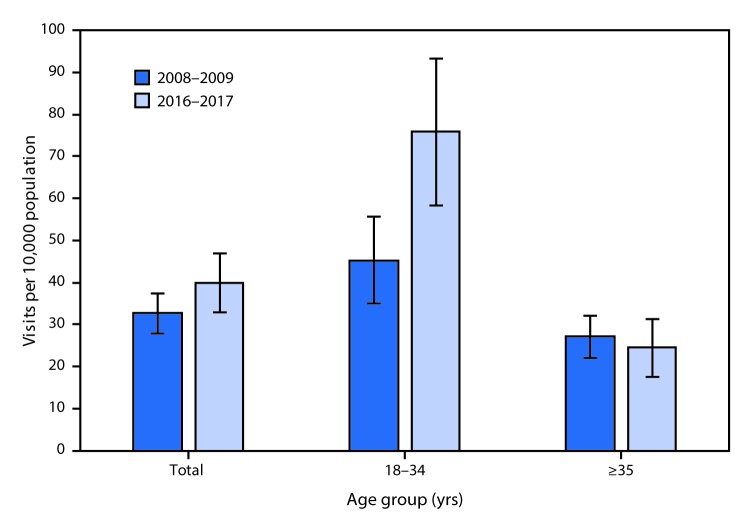
The rate of ED visits with a primary diagnosis or primary complaint of substance abuse or dependence by patients aged 18–34 years in the United States increased from 45.4 visits per 10,000 persons in 2008–2009 to 76.0 visits in 2016–2017 but remained stable among patients aged ≥35 years (27.2 in 2008–2009 and 24.6 in 2016–2017). In both periods, persons aged 18–34 years were more likely to visit the ED for substance abuse or dependence than those aged ≥35 years.

**Source:** National Hospital Ambulatory Medical Care Survey, 2008–2017.

